# A Novel Preparation Method for 5-Aminosalicylic Acid Loaded Eudragit S100 Nanoparticles

**DOI:** 10.3390/ijms13056454

**Published:** 2012-05-24

**Authors:** Daode Hu, Liang Liu, Wenjuan Chen, Sining Li, Yaping Zhao

**Affiliations:** 1 Department of Clinical Pharmacology, Shanghai First People’s Hospital, Medical College, Shanghai Jiao Tong University, Shanghai 200080, China; E-Mails: callenliu@yahoo.cn (L.L.); chenwenjuan1986@126.com (W.C.); 2 College of Chemistry and Chemical Engineering, Shanghai Jiao Tong University, Shanghai 200240, China; E-Mail: l5011129012@hotmail.com

**Keywords:** supercritical antisolvent, solution enhanced dispersion by supercritical fluids, Eudragit S100, 5-ASA, nanoparticles, colon-specific

## Abstract

In this study, solution enhanced dispersion by supercritical fluids (SEDS) technique was applied for the preparation of 5-aminosalicylic acid (5-ASA) loaded Eudragit S100 (EU S100) nanoparticles. The effects of various process variables including pressure, temperature, 5-ASA concentration and solution flow rate on morphology, particle size, 5-ASA loading and entrapment efficiency of nanoparticles were investigated. Under the appropriate conditions, drug-loaded nanoparticles exhibited a spherical shape and small particle size with narrow particle size distribution. In addition, the nanoparticles prepared were characterized by X-ray diffraction, Differential scanning calorimetry and Fourier transform infrared spectroscopy analyses. The results showed that 5-ASA was imbedded into EU S100 in an amorphous state after SEDS processing and the SEDS process did not induce degradation of 5-ASA.

## 1. Introduction

Colon-specific drug delivery system can be applied in many therapeutic areas, especially in the treatment of inflammatory bowel disease (IBD) [1–4], which is characterized by recurrent, idiopathic inflammatory disorders involving the mucosa and submucosa of the colon. 5-aminosalicylic acid (5-ASA), a typical anti-inflammatory drug, has been used for over 30 years in the treatment of IBD. After the oral administration of free 5-ASA, it is readily absorbed in the upper part of the gastrointestinal tract and then extensively metabolized to N-acetyl-5-ASA which is inactive in treating IBD [5,6]. The clinical efficacy of 5-ASA depends on sufficient therapeutical concentration of the drug at the site of the colon mucosa. Therefore, it is preferable for treatment of IBD to deliver the 5-ASA specifically to the colon.

Although many efforts have been made for a higher specificity of entrapped active compounds release by designing new drug delivery devices for the treatment of IBD [7,8], all marketed delivery systems still appear to be insufficiently selective [9]. This is mainly due to the fact that the drug release mechanisms are based on physiological parameters such as pH, bacterial and time which are not related to the inflammation and barely to its location [6,9,10]. Nanoparticles have shown their specific accumulation in the inflamed tissues in the colon. The accumulation phenomenon of nanoparticles is observed to be particle size dependent with an increased adhesiveness for smaller particle diameters [11], which may therefore allow a selective delivery to the site of inflammation for the treatment of IBD. Indeed, nanoparticles showed promising results and demonstrated their therapeutic potential [6,10,12–15].

Recently, the application of supercritical antisolvent (SAS) technique in the field of particle formation, encapsulation and impregnation has received increasing attention, and is anticipated to become a very attractive alternative to many conventional processes [16–19]. Compared with most traditional processes, such as milling, spray-drying, recrystallization using solvent evaporation and liquid antisolvents, the SAS technique has major advantages because of its lower residual solvents, simpler steps and mild operation temperatures. Besides, encapsulation by means of supercritical fluid is of great advantage because of its ability to produce uniform particle size and controlled morphology [16]. Solution enhanced dispersion by supercritical fluids (SEDS) is one of the SAS processes, which was developed to achieve small droplet size and intense mixing of supercritical fluids with solution for increasing mass transfer rate at the interface via coaxial nozzle [20].

The objective of this study was to prepare the 5-ASA loaded colon-specific nanoparticles using pH-sensitive polymer Eudragit S100 (EU S100) via SEDS technique and to study the effects of various process parameters on the morphology, particle size, 5-ASA loading and entrapment efficiency of nanoparticles. EU S100 is an anionic copolymer based on methacrylic acid and methyl methacrylate (1:2 ratio). It is insoluble in acids and pure water, whereas soluble in aqueous solution at pH 7 or higher. When used to entrap 5-ASA, it is expected to protect 5-ASA from degradation and allow it to be released in the region of colon. Moreover, the nanoparticles obtained were characterized by Scanning electron microscopy (SEM), Laser particle size analyzer, High efficiency liquid chromatography (HPLC), X-ray diffraction (XRD), Differential scanning calorimetry (DSC) and Fourier transform infrared (FTIR) spectroscopy.

## 2. Results and Discussion

### 2.1. Effect of Process Parameters

In all trials, the mixed organic solvents of acetone and DMSO (7:3, v/v) were used as dissolvent of 5-ASA and EU S100. Commonly, a critical factor in the SEDS process is the selection of appropriate organic solvent for nanoparticle preparation. 5-ASA, a hydrophilic compound, is mostly insoluble in most of organic solvents. Supercritical fluid miscible organic solvents such as dimethyl sulfoxide (DMSO) are suitable for dissolving these hydrophilic compounds [16]. The choice of acetone is rational as it served as the solvent for EU S100, and has always been used for nanoparticle preparation via SAS process [21–23]. The determination of the ratio between DMSO and acetone was based on the following principles: at first, the organic solvent with high volatility which can induce high volume of expansion and can easily be removed from the system should be selected; the solubility of the polymer in the organic solvent should be higher than that of the drug in the solvent, which favors first precipitates of the drug, then drug is coated by the polymer, and finally drug encapsulation by the polymer occurs [16]. Both principles favors for a large proportion of acetone to DMSO. Therefore, the mixed solvents of acetone and DMSO (7:3, v/v) were used as the organic solution in SEDS process through the preliminary experiments (data not shown).

The experimental conditions and part results were summarized in Table 1, from which it can be seen that the process parameters had impact on the morphology, mean particle size, 5-ASA loading and entrapment efficiency.

#### 2.1.1. Pressure Effect

**Figure 1:**
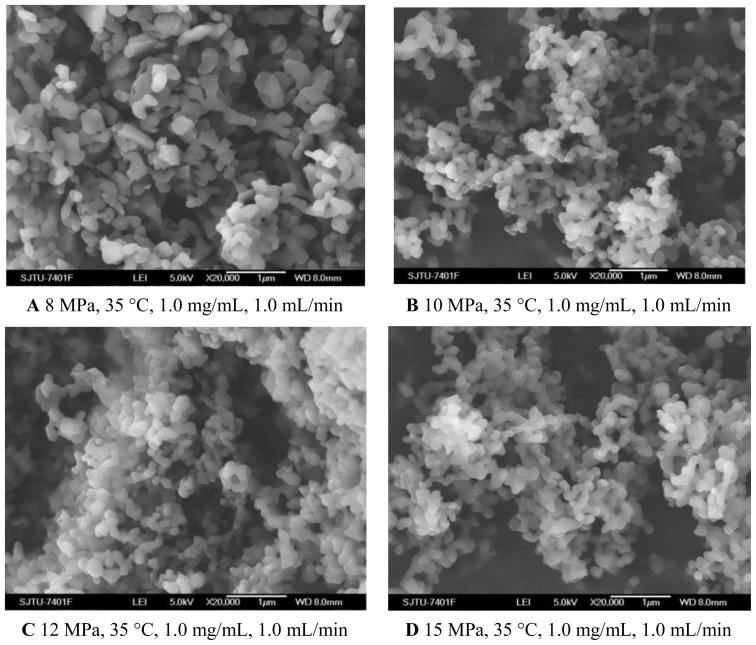
SEM images of nanoparticles prepared by SEDS process with different pressures. **A** 8 MPa, 35 °C, 1.0 mg/mL, 1.0 mL/min **B** 10 MPa, 35 °C, 1.0 mg/mL, 1.0 mL/min **C** 12 MPa, 35 °C, 1.0 mg/mL, 1.0 mL/min **D** 15 MPa, 35 °C, 1.0 mg/mL, 1.0 mL/min

**Figure 2:**
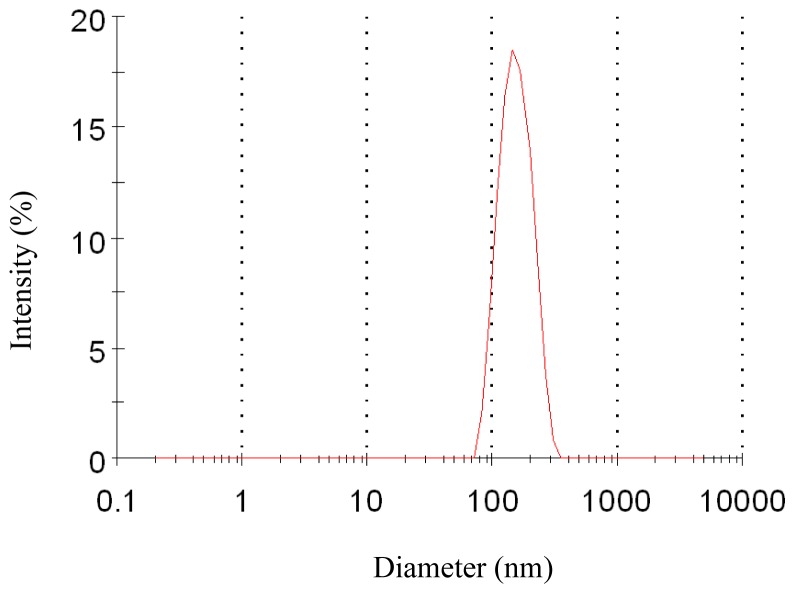
Particle size distribution of nanoparticles (12 MPa, 35 °C, 1.0 mg/mL, 1.0 mL/min).

To evaluate the effect of pressure on the characteristics of the nanoparticles, experiments were carried out changing the pressure from 8 MPa to 15 MPa. The effect of pressure on morphology and mean particle size of nanoparticles was illustrated in Figure 1A–D and Table 1, respectively. One pattern of the particle size distribution was shown in Figure 2. It was noticed that at pressure of 10 MPa, 12 MPa and 15 MPa, the morphology of nanoparticles exhibited spherical shape with smooth surface, while at the pressure of 8 MPa, the morphology became irregular. Nanoparticles could be produced when the pressure was well above the mixture critical point (MCP). In the CO_2_-DMSO-acetone system, the MCP of this system is above 8 MPa as described previously [24]. The experimental condition of 8 MPa was located in the two-phase region, which might be a reason that the nanoparticles could not be formed. However, the experimental conditions of 10 MPa, 12 MPa and 15 Mpa were located in the supercritical region and were far above the MCP pressure, so the nanoparticles with spherical shape could be formed. Additionally, the particle size was decreased with a pressure increase from 10 MPa to 15 MPa. This could be explained in terms of the volumetric expansion of the liquid phase [25]. With increasing the pressure at a fixed temperature, the solubility of mixed solvent in the CO_2_ will increase [24]. More CO_2_ will diffuse into the mixed solvent, thus causing higher supersaturation ratio of the expanded liquid solution, which subsequently resulted in the formation of smaller particles. From Table 1, it could be found that the higher pressure favored lower 5-ASA loading and higher entrapment efficiency. The solubility of 5-ASA in mixed solvents of supercritical CO_2_ (scCO_2_) + acetone + DMSO was increased as the solvent power of the scCO_2_ was raised with increasing pressure at a fixed temperature [26]. Then, less 5-ASA precipitated, causing lower 5-ASA loading. And more 5-ASA on the surface of samples might be dissolved in the scCO_2_ and be taken away, then resulting in higher entrapment efficiency.

#### 2.1.2. Temperature Effect

**Figure 3:**
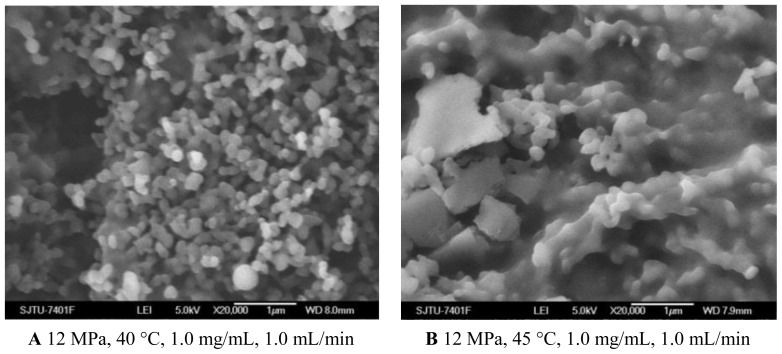
SEM images of nanoparticles prepared by SEDS process with different temperatures. **A** 12 MPa, 40 °C, 1.0 mg/mL, 1.0 mL/min **B** 12 MPa, 45 °C, 1.0 mg/mL, 1.0 mL/min

As shown in Figures 1C and 3A and Table 1, the morphology of nanoparticles changed from the spherical shape with smooth surface at 35 °C to irregular surface at 40 °C, despite the fact that the mean particle size of nanoparticles increased a little. Moreover, when the temperature increased to 45 °C, the aggregation was serious, as shown in Figure 3B. This might be due to more polymer plasticization effect of CO_2_ [27]. Bleich *et al*. [28] reported that the particles produced at higher temperatures lead to compact agglomerates by bridging. 5-ASA loading was found decreased with increasing temperature. From the point view of thermodynamics, in the SAS process, if the solvent could not be completely miscible with the scCO_2_, two fluid phases would be formed and the solute would still be dissolved or partly dissolved in the liquid-rich phase [29]. It might be easier for scCO_2_ and the organic solvents to form one phase at the lower temperature of 35 °C and 40 °C. In that case, the higher solvent power of scCO_2_ would extensively decrease solubility of 5-ASA in the mixed solvents, leading to more 5-ASA precipitated. The entrapment efficiency was found decreased with higher temperature, which might due to more 5-ASA would still be attached on the surface of the nanoparticles as the solubility of 5-ASA was decreased in scCO_2_.

#### 2.1.3. Effect of 5-ASA Concentration

**Figure 4:**
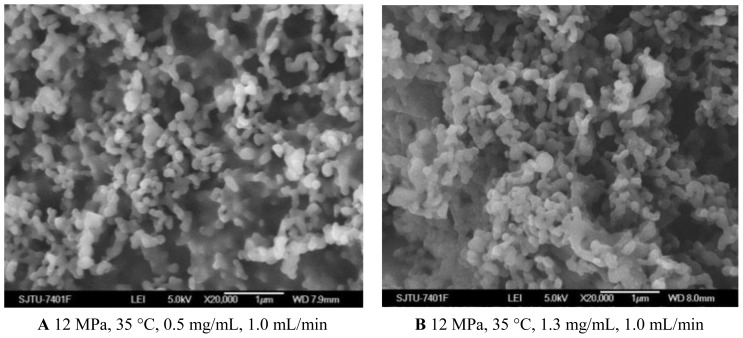
SEM images of nanoparticles prepared by SEDS process with different 5-ASA concentrations.

From the Figures 1C and 4A, B and Table 1, it could be concluded that the lower concentration of 5-ASA was beneficial for spherical shape and smaller particle size of nanoparticles. This phenomenon has been explained in terms of nucleation and growth processes [30,31]. In the case of lower concentration, saturation and then precipitation of the drug occur very late during the droplet expansion process. Therefore, nucleation is the prevailing mechanism, producing smaller particles. 5-ASA loading increased because of sufficient quantities of 5-ASA precipitated with increasing the concentration of 5-ASA. Entrapment efficiency decreased with the higher concentration of 5-ASA, which might be attributed to, at a higher concentration of the drug, the amount of polymer not being sufficient to effectively encapsulate the drug [32,33].

#### 2.1.4. Effect of Solution Flow Rate

**Figure 5:**
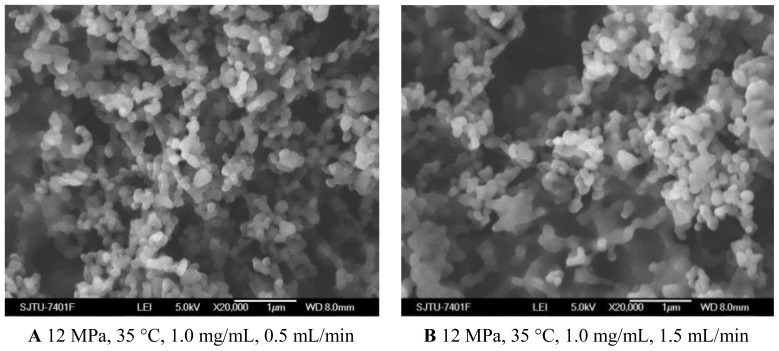
SEM images of nanoparticles prepared by SEDS process with different solution flow rates.

As shown in Figures 1C and 5A, B, when the solution flow rate increased from 0.5 mL/min to 1.5 mL/min, the morphology of the nanoparticles became aggregated, particularity at the flow rate of 1.5 mL/min. It was also known from the Table 1 that the mean particle size of nanoparticles increased with an increase of solution flow rate, and the solution flow rate significantly affected the drug loading and entrapment efficiency. These facts might be related to hydrodynamics aspects. With an increase of the solution flow rate, the kinetic energy per unit mass of liquid gaining from scCO_2_ is decreased, which decreases the interaction of antisolvent and solution, hence leading to the change of the morphology, size of particles, 5-ASA loading and entrapment efficiency [25,34].

### 2.2. Physicochemical Properties of Nanoparticles

#### 2.2.1. XRD Analysis

**Figure 6:**
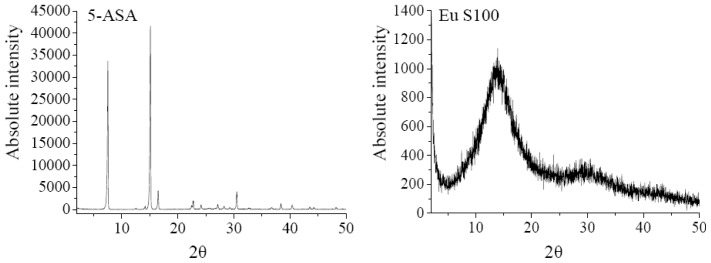

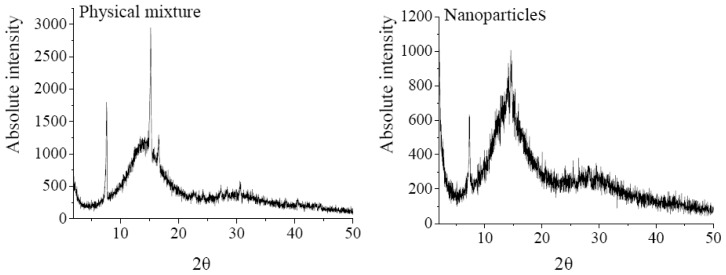
XRD patterns of 5-ASA, EU S100, physical mixture and nanoparticles (12 MPa, 35 °C, 1.0 mg/mL, 1.0 mL/min).

The XRD analysis was performed to investigate the crystalline structure change of nanoparticles after SEDS process. The XRD patterns of 5-ASA, EU S100, physical mixture of 5-ASA and EU S100, and 5-ASA/EU S100 nanoparticles were shown in Figure 6. The physical mixture was prepared by mixing 5-ASA and EU S100 directly, in which the content of 5-ASA was the same as that of nanoparticles (7.2%, w/w). As shown in Figure 6, the diffraction angels of 2θ = 7.54°, 15.12°, 16.5°, 30.52 °C can be regarded as the feature crystalline peaks of 5-ASA. It could be seen that the pattern of the physical mixture was simply a superimposition of the patterns of the two raw materials, while the pattern of nanoparticles was significantly different from that of the physical mixture, *i.e*., only one diffraction peak of 5-ASA at 7.54° was present, which implies a reduction in crystallinity of the treated 5-ASA as a result of SEDS processing. Reverchon *et al*. [35] suggested that the very fast precipitation process in supercritical conditions does not allow the organization of the compound in a regular crystalline form. This is also consistent with the results reported by Zu *et al*. [18].

#### 2.2.2. DSC Analysis

**Figure 7:**
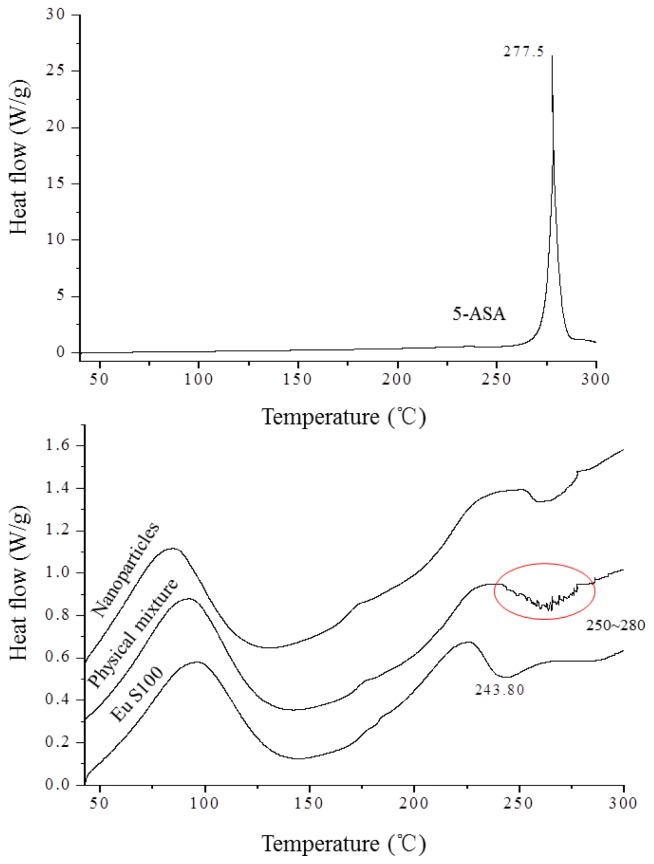
DSC thermograms of 5-ASA, EU S100, physical mixture and nanoparticles (12 MPa, 35 °C, 1.0 mg/mL, 1.0 mL/min).

The DSC technique is one of the most convenient methods for investigating the compatibility of polymer blends, therefore it was used to investigate thermodynamic compatibility between 5-ASA and EU S100 in their nanoparticles based on crystalline melting temperature and the glass transition temperature. Figure 7 depicted the DSC thermograms of above samples. In the Eu S100, a glass transition temperature region and an exothermic peak located at 243.8 °C were observed. The raw 5-ASA showed a sharp endothermic peak at 277.5 °C that corresponded to its melting point, indicating its crystalline nature. A physical mixture of the drug with polymer resulted in the disappearance of such a fusion peak, replaced by broad endothermic signals exhibiting a reduced melting endotherm in the range of 250 °C to 280 °C. The presence of endothermic signals in the physical mixture confirmed that 5-ASA crystals still exist in physical mixture [36,37]. This type of interaction was previously observed in the physical mixture of piroxicam with Eu S100 and diflunisal with Eudragit RL 100 [37,38]. However, for nanoparticles, the intensity of melting peak of 5-ASA disappeared completely. The disappearance of the drug endothermic peak in the nanoparticles suggested that 5-ASA might be imbedded into EU S100 and existed in an amorphous state in the nanoparticles, indicating a thermodynamic compatibility between 5-ASA and EU S100 [26].

#### 2.2.3. FTIR Spectroscopy Analysis

**Figure 8:**
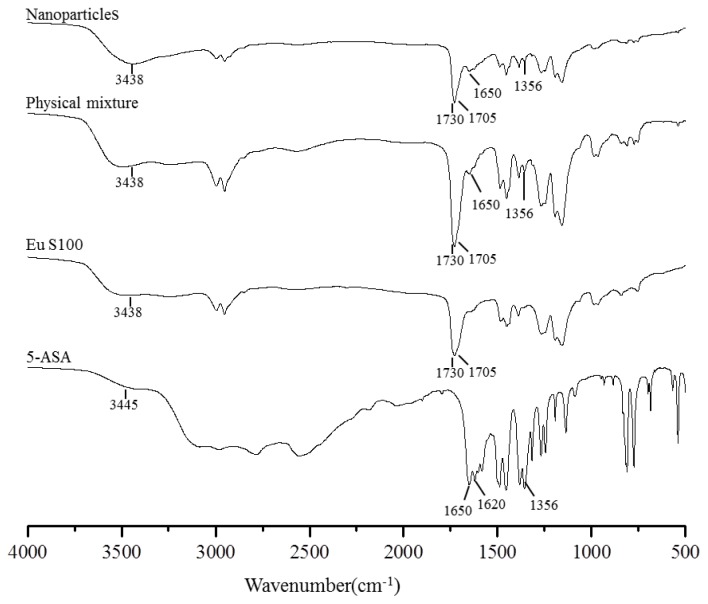
FTIR spectra of 5-ASA, EU S100, physical mixture and nanoparticles (12 MPa, 35 °C, 1.0 mg/mL, 1.0 mL/min).

The FTIR spectroscopy analysis was used to obtain information on the change of chemical structure after SEDS processing, and the FTIR spectra of above samples were shown in Figure 8. Some characteristic functional group and wavenumber were summarized in Table 2. In the FTIR spectra of EU S100, the C=O vibration band of the carboxylic groups presented as a shoulder at 1705 cm^−1^; whereas the peak at 1730 cm^−1^ was attributed to the esterified carboxyl groups [38]. The characteristic peaks of 5-ASA spectra had been already described by Mladenovska *et al*. [39], and they corresponded to: 3445 cm^−1^ (mutual overlapping of νNH and νOH stretching), 1650 cm^−1^ (C=O stretch), 1620 cm^−1^ (NH blend), 1356 cm^−1^ (C–N stretch), 2000–3000 cm^−1^ (stretching vibrations of the hydrogen bonds). The FTIR spectra of physical mixture and nanoparticles were similar to the EU S100 spectra, except for the 5-ASA peak of C–N located at 1356 cm^−1^ and C=O located at 1650 cm^−1^. The reason might be that the amount of 5-ASA in both physical mixture and nanoparticles was relatively low, resulting in the most characteristic peaks of 5-ASA occurred covered by broad peak from EU S100 in this region. The FTIR spectra between physical mixture and nanoparticles didn’t show any significant differences, indicating the molecular structure of processed nanoparticles has no change, as confirmed by HPLC analysis (data not shown).

## 3. Experimental Section

### 3.1. Materials

5-ASA (98% purity) was purchased from Shanghai Chemical Reagent Plant (Shanghai, China). EU S100 polymer was kindly donated from Evonik Pharma Polymers (Darmstadt, Germany). CO_2_ (99.95% purity) was obtained from SJTU Chemical Store (Shanghai, China). Acetone (≥99.5% purity), Dimethyl sulfoxide (DMSO, 98.5% purity) were purchased from Shanghai Lingfeng Chemical Reagent Co., Ltd. (Shanghai, China). All solutions used in HPLC analysis were of HPLC grade and filtered using a 0.22 μm membrane filter with a filtration system (SHB-III, China).

### 3.2. Experimental Apparatus and Procedure

The experimental apparatus was purchased from Nantong Huaxing Petroleum Devices Co. Ltd. (Jiangsu, China). The technical details of the SEDS process in this study had already been described in our previous work [25,28]. Briefly, scCO_2_ and organic solution were separately pumped into a high-pressure vessel (200 mL) through different inlets of a coaxial nozzle. The coaxial nozzle was located on the top of the high-pressure vessel, with an inner tubule (diameter is 0.2 mm) and an outer tubule (diameter is 1 mm). A rapid mutual mixing diffusion at the outlet interface of scCO_2_ and the organic solution occurred instantaneously. Meanwhile, solutes dissolved originally in organic solvent reached its supersaturation in very short time, resulting in the formation of solid nanoparticles in the vessel. Once feeding the solution finished, the scCO_2_ was continuously pumped for about 45 min in order to remove the residual organic solvent of the samples. Finally, the vessel was slowly depressurized to the atmospheric pressure and the samples were collected for further characterization.

### 3.3. Preparation Conditions of 5-ASA/EU S100 Nanoparticles

The experimental conditions were designed as shown in Table 1. In all trials, the mixed organic solvents of acetone and DMSO (7:3, v/v) were used for dissolving 5-ASA and EU S100, in which the EU S100 concentration was fixed at 4.0 mg/mL, the flow rate of scCO_2_ was maintained at 3 kg/h. The process parameters studied were varied in the range as presented in Table 1 to determine their effects on characteristics of nanoparticles precipitated. In our study, only one process parameter was varied according to the range specified while other operating conditions were kept unchanged.

### 3.4. Nanoparticle Characterization

#### 3.4.1. SEM Analysis

The surface morphology of the processed samples was observed by SEM (JEOL, JSM-7401F, Japan). In the analysis, the samples were firstly attached to a small piece of electro-conductive paste silicon chip, then sputter-coated with Au using an LDM-1500 vacuum sputter coater (Shanghai Electron Optical Tech, Academe PR China).

#### 3.4.2. Particle Size and Particle Size Distribution Analysis

The particle size and particle size distribution were analyzed by a laser particle size analyzer (ZS90, Malvern, UK). Before measurement, a small amount of nanoparticles were suspended in distilled water, the suspension was then dispersed by ultrasonic waves with power 120 W for 1 min. Particle size and particle size distribution were then assayed by the analytic software. The measurement was performed in triplicate. PSD is expressed by span, which can be calculated according to the following Equation:

(1) $$\text{Span}=\frac{d_{0.9}-d_{0.1}}{d_{0.5}}$$

where *d*_0.1_, *d*_0.5_, and *d*_0.9_ are the volumes occupied by particles 10% undersize diameter, mean size diameter, and 90% undersize diameter, respectively.

#### 3.4.3. 5-ASA Loading and Entrapment Efficiency Measurement

The content of 5-ASA was measured by HPLC (Agilent 1100 Series HPLC system, Agilent Technologies Shanghai, China). Agilent Eclipse XDB-C18 4.6 mm × 250 mm × 5 μm column was used and an ultraviolet detector was set at 240 nm. The mobile phase consisted of mobile phase A (dissolving and diluting tetrabutylammonium hydrogen sulfate 3.4 g, sodium acetate 1.4 g to 1000 mL with purified water, then adjusting with 1 M sodium hydroxide to a pH of 6.6) and mobile phase B (acetonitrile) (77:23 v/v). The volumetric flow was 1.0 mL/min and the temperature of column was maintained at 25 °C.

The 5-ASA loading was defined as the content of 5-ASA in the nanoparticles. The measurement method was as follows: a known quantity of nanoparticles was weighed, and then dissolved in 0.1 M phosphate-buffered saline (PBS, pH 7.4), in which the amount of 5-ASA was determined as described above in triplicate. Entrapment efficiency was defined as the amount of 5-ASA entrapped inside the nanoparticles and measured as follows: a certain amount of the nanoparticles was firstly dispersed in 3 mL HCL (0.1 M, pH 1.2) and vortexed for 1 min, and then centrifuged at 13,000× g for 3 min. The supernatant was collected and the precipitate was redispersed with 3 mL HCL. The same step was repeated three times to make sure that the 5-ASA attached on the surface was all collected. The amount of 5-ASA in the total collected solution was determined as given above in triplicate. The equations of the 5-ASA loading and entrapment efficiency were calculated by Equations 2 and 3, respectively.

(2) $$5-\text{ASA loading }(\%)=\frac{\text{Total amount of }5-\text{ASA in nanoparticles}}{\text{Total amount of nanoparticles}}\times 100$$

(3) $$\text{Entrapment efficiency }(\%)=\frac{\begin{array}{l} \text{Total amount of }5-\text{ASA in nanoparticles}-\text{Amount}\;\text{of }5-\text{ASA on the surface of nanoparticles} \end{array}}{\text{Total amount of }5-\text{ASA in nanoparticles}}\times 100$$

#### 3.4.4. XRD Analysis

The crystalline characters of 5-ASA, EU S100, physical mixture of 5-ASA and EU S100, and 5-ASA/EU S100 nanoparticles were analyzed by XRD (D/max-2200/PC, Japan Rigaku Corporation, Japan) with a rotating Cu anode. The Cu Ka radiation was generated at 20 mA and 40 kV and monochromatized by a nickel filter. Diffraction patterns recorded the X-ray intensity as a function of 2θ angle covering from 2.0° to 50.0°. The scanning rate was 6°/min.

#### 3.4.5. DSC Analysis

DSC (Pyris 1, Perkin Elmer, Inc., USA) was used to investigate the thermal behaviors of the samples above. 5-mg samples were placed in aluminum pans and sealed. The probes were heated from 40 °C to 300 °C at a rate of 10 °C/min under nitrogen atmosphere.

#### 3.4.6. FTIR Spectroscopy Analysis

FTIR (Spectrum 100, Perkin Elmer, Inc., USA) spectroscopy analysis was used to obtain information on the change of chemical structure of samples above. The sample powder was dispersed in KBr powder and this mixture was pressed into a pellet for scanning in the infrared range of 500–4000 cm^−1^.

## 4. Conclusions

In the present work, a process for preparation of 5-ASA loaded Eu S100 nanoparticles by SEDS technique has been developed. The influences of various process parameters on morphology, particle size, 5-ASA loading and entrapment efficiency were systematically investigated. The morphology and particle size of the nanoparticles could be adjusted by modifying various process parameters, such as pressure, temperature, 5-ASA concentration and solution flow rate. With the appropriate parameters, nanoparticles with spherical shape and smaller particle size could be obtained. The lower temperature, 5-ASA concentration and solution flow rate coupled with higher pressure favored smaller and more regular spherical nanoparticles. The 5-ASA loading and entrapment efficiency were also dependent on different process parameters and it was found that the solution flow rate significantly affect the drug loading and entrapment efficiency. The XRD and DSC analyses revealed that 5-ASA was imbedded into EU S100 and existed in an amorphous state in the nanoparticles. FTIR analysis showed that the SEDS process did not induce degradation of 5-ASA. These results collectively indicate that the SEDS technique is a simple and effective process for preparing nanoparticles.

## Figures and Tables

**Table 1:** Experiment conditions and partial results of solution enhanced dispersion by supercritical fluids (SEDS) process.

Pressure MPa	Temperature °C	5-ASA concentration mg/mL	Solution flow rate mL/min	5-ASA loading % a	Entrapment efficiency % a	Mean particle size nm a	Span a
8	35	1.0	1.0	12.78 ± 0.28	18.90 ± 1.20	395 ± 89	1.98 ± 0.25
10	35	1.0	1.0	13.06 ± 0.86	37.26 ± 0.98	192 ± 30	1.05 ± 0.12
12	35	1.0	1.0	7.21 ± 0.26	47.93 ± 1.36	159 ± 22	0.81 ± 0.05
15	35	1.0	1.0	3.35 ± 0.21	51.12 ± 1.85	147 ± 16	0.62 ± 0.05
12	40	1.0	1.0	6.20 ± 0.08	46.63 ± 0.48	169 ± 26	1.14 ± 0.93
12	45	1.0	1.0	4.16 ± 0.35	30.36 ± 0.35	aggregated	-
12	35	0.5	1.0	3.49 ± 0.08	60.51 ± 0.21	137 ± 20	0.67 ± 0.04
12	35	1.3	1.0.	9.87 ± 0.35	36.45 ± 1.24	257 ± 40	1.83 ± 0.20
12	35	1.0	0.5	21.91 ± 0.56	39.48 ± 1.54	137 ± 16	0.78 ± 0.05
12	35	1.0	1.5	5.99 ± 0.32	3.21 ± 0.38	227 ± 48	1.57 ± 0.15

a Experimental data are expressed as mean ± SD (*n* = 3).

**Table 2:** Shows wavenumber (cm^−1^) and functional group for 5-ASA, Eu S100, physical mixture and nanoparticles (12 MPa, 35 °C, 1.0 mg/mL, 1.0 mL/min) using FTIR.

Eu S100		5-ASA		Physical mixture		Nanoparticles	
			
Wavenumber (cm^−1^)	Functional group	Wavenumber (cm^−1^)	Functional group	Wavenumber (cm^−1^)	Functional group	Wavenumber (cm^−1^)	Functional group
3438	O–H	3445	N–H	3438	O–H	3438	O–H
1705	C=O	3445	O–H	1705	C=O	1705	C=O
1730	C=O	1650	C=0	1730	C=O	1730	C=O
		1620	N–H	1650	C=0	1650	C=0
		1356	C–N	1356	C–N	1356	C–N
